# Histone acetylation mediates epigenetic regulation of transcriptional reprogramming in insects during metamorphosis, wounding and infection

**DOI:** 10.1186/1742-9994-9-25

**Published:** 2012-10-04

**Authors:** Krishnendu Mukherjee, Rainer Fischer, Andreas Vilcinskas

**Affiliations:** 1 Department of Bioresources, Fraunhofer Institute of Molecular Biology and Applied Ecology, Winchester Str. 2, Giessen, 35395, Germany; 2 Institute of Phytopathology and Applied Zoology, Justus-Liebig-University of Giessen, Heinrich-Buff-Ring 26-32, Giessen, 39592, Germany

**Keywords:** Epigenetics, Histone acetylation, Development, Metamorphosis, Immunity, *Galleria mellonella*

## Abstract

**Background:**

Gene expression in eukaryotes is regulated by histone acetylation/deacetylation, an epigenetic process mediated by histone acetyltransferases (HATs) and histone deacetylases (HDACs) whose opposing activities are tightly regulated. The acetylation of histones by HATs increases DNA accessibility and promotes gene expression, whereas the removal of acetyl groups by HDACs has the opposite effect.

**Results:**

We explored the role of HDACs and HATs in epigenetic reprogramming during metamorphosis, wounding and infection in the lepidopteran model host *Galleria mellonella*. We measured the expression of genes encoding components of HATs and HDACs to monitor the transcriptional activity of each enzyme complex and found that both enzymes were upregulated during pupation. Specific HAT inhibitors were able to postpone pupation and to reduce insect survival following wounding, whereas HDAC inhibitors accelerated pupation and increased survival. The administration of HDAC inhibitors modulated the expression of effector genes with key roles in tissue remodeling (matrix metalloproteinase), the regulation of sepsis (inhibitor of metalloproteinases from insects) and host defense (antimicrobial peptides), and simultaneously induced HAT activity, suggesting that histone acetylation is regulated by a feedback mechanism. We also discovered that both the entomopathogenic fungus *Metarhizium anisopliae* and the human bacterial pathogen *Listeria monocytogenes* can delay metamorphosis in *G. mellonella* by skewing the HDAC/HAT balance.

**Conclusions:**

Our study provides for the first evidence that pathogenic bacteria can interfere with the regulation of HDACs and HATs in insects which appear to manipulate host immunity and development. We conclude that histone acetylation/deacetylation in insects mediates transcriptional reprogramming during metamorphosis and in response to wounding and infection.

## Background

Gene expression in eukaryotes is regulated by epigenetic mechanisms such as histone acetylation and deacetylation, which modify chromatin structure and alter the accessibility of DNA to transcription factors. The transfer of acetyl groups to and from histones is controlled by histone acetyltransferases (HATs) and histone deacetylases (HDACs), which have opposing activities. The acetylation of histones by HATs increases DNA accessibility and therefore promotes gene expression, whereas HDACs reduce access to DNA and therefore suppress gene expression. In humans, HAT and HDAC activities are tightly regulated to maintain a productive balance, and changes in this equilibrium have been shown to cause both developmental and immunological defects [1-3]. Mediators of histone acetylation are evolutionarily conserved between mammals and insects [4,5].

Consequently, we predicted that HAT and HDAC inhibitors should have opposite effects on insect development and pathogenesis if these processes could be studied in the same model system. We therefore used the larvae of the greater wax moth *Galleria mellonella*, which provide a useful developmental model, a powerful model host for human pathogens [6,7] and a source of drug candidates against them [8]. For example, *G. mellonella* has recently been established as a model for the investigation of *Listeria monocytogenes* pathogenesis and as a source of antibiotics that help to prevent the resulting food-borne disease [9,10].

Previous studies in *G. mellonella* have identified molecular mechanisms that influence development and immunity, indicating that this species is suitable for the investigation of epigenetic regulation [11-13]. We recently analyzed the *G. mellonella* transcriptome during metamorphosis and/or following challenge with bacterial lipopolysaccharide (LPS) and identified several differentially-expressed genes encoding components of HATs and HDACs [14]. This suggested that HATs and HDACs are intimately involved in the control of transcriptional remodeling during metamorphosis and infection, and may regulate the injury-induced expression of genes encoding products such as the antimicrobial peptide galiomycin [15], the inhibitor of metalloproteinases from insects (IMPI), which protects against sepsis [16,17], mitogen-activated protein (MAP) kinase, which is involved in immunity-related signaling, and a matrix metalloproteinase involved in tissue remodeling during metamorphosis and infections [18,19].

Here we show that infection with virulent pathogens (*Listeria monocytogenes* or the entomopathogenic fungus *Metarhizium anisopliae*) postponed the formation of pupae, whereas non-pathogenic *Escherichia coli* accelerated the onset of metamorphosis, with concomitant effects on downstream effector genes. The impact of these data on our current understanding of the epigenetic control of development and immunity in insects is discussed.

## Results

### Expression of genes encoding HATs and HDACs during pupation

**Figure 1:**
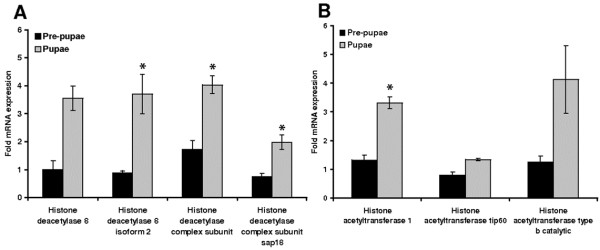
**Transcriptional analysis of genesencoding HDACs and HATsin *G. mellonella *pre-pupae and pupae.** The transcription of (**A**) *histone deacetylase 8*, *histone deacetylase 8 isoform2*, *histone deacetylase complex* and *histone deacetylase complex subunitsap 18*, and (**B**) *histone acetyltransferase 1*, *histone acetyltransferase tip 60*, and *histone acetyltransferase type bcatalytic subunit* in pre-pupae and pupae were determined by quantitative real time RT-PCR relative to the expression levels in last-instar *G. mellonella* larvae. Values were normalized against the 18S rRNA housekeeping gene and represent means of three independent measurements ± standard deviations (*, *p* < 0.05).

Our comprehensive transcriptomic analysis of *G. mellonella* revealed many genes that are differentially expressed during metamorphosis and in response to injected bacterial lipopolysaccharides [14], including four genes encoding HDACs (*HDAC8*, *HDAC8 isoform 2*, *HDAC complex subunit* and *HDAC complex subunit sap18*) and three encoding HATs (*HAT1*, *HAT tip60* and *HAT type b catalyticsubunit*). Based on the resulting sequence data, we designed real-time RT-PCR primers to determine the expression levels of these genes in last-instar larvae, prepupae and early pupae (Table 1). We found that genes encoding HDACs and histone acetyltransferase 1 were significantly expressed at higher levels in pupae than in last-instar larvae and pre-pupae, indicating transcriptional reprogramming associated with metamorphosis (Figures 1A-B). The simultaneous upregulation of HDAC and HAT genes suggests that normal development requires finely balanced enzyme activities, and that the disruption of this balance would interfere with metamorphosis.

**Table 1:** **Primer sequences used forRT PCR**

	**Genes**	**Primer sequences**
1	histone deacetylase 8-forward	5`-GATACAGTGTGGTGCGGATG-3`
	histone deacetylase 8-reverse	5`-GCAACAAGAGCAGTGATGGA-3`
2	histone deacetylase 8 isoform 2-forward	5`- TCTTCATCTTGTGGGGTTGA -3`
	histone deacetylase 8 isoform 2-reverse	5`- GCGGGCTTCTTTAATACACG -3`
3	histone deacetylase complex subunit-forward	5`- ACTTCAGGCGAGTCCATCAG -3`
	histone deacetylase complex subunit-reverse	5`- ACAACGAACGTTGCAGACAG -3`
4	histone deacetylase complex subunit sap18-forward	5`- GAAACTCGACGCAAAGGAAC -3`
	histone deacetylase complex subunit sap18-reverse	5`- CTCATTGGTGGAGGCATTCT -3`
5	histone acetyltransferase 1- forward	5`- CGCATTGTGCCATTTAGTTG -3`
	histone acetyltransferase 1- reverse	5`- TGAAGGCTTCCTGCACTGTA -3`
6	histone acetyltransferase tip60- forward	5`- CGCGAAATGGTAACAAACAG-3`
	histone acetyltransferase tip60- reverse	5`- TGGAGAGCCACATAACAACTG -3`
7	histone acetyltransferase type b catalytic- forward	5`- CCTGAACGTTGTGGACATCA -3`
	histone acetyltransferase type b catalytic- reverse	5`- CGCGCCTGTTTCTTGTTTAT -3`
8	MMP-I-forward	5′-CGCAGAGACGTGGACTATCA-3′
	MMP-I-reverse	5′-CATAAGGGCAGAGCGAACAT-3′
9	IMPI-forward	5′-AGATGGCTATGCAAGGGATG-3′
	IMPI-reverse	5′-AGGACCTGTGCAGCATTTCT-3′
10	p38 MAP kinase- forward	5’-CTGATGGCAAGAGGATTCG-3′
	p38 MAP kinase- reverse	5’-CTTGGGACGCCTAGTCAGG-3′
11	Galiomycin-forward	5′-GGA TCC ATG GCG AAA AATTTC CAG TCC-3′
	Galiomycin-reverse	5′-GTC GAC TTA CTCGCA CCA ACA ATT GAC GTT-3′
12	18S- forward rRNA	5′-ATGGTTGCAAAGCTGAAACT-3′
	18S- Reverse rRNA	5′TCCCGTGTTGAGTCAAATTA-3′

### Effects of HAT and HDAC inhibitors on development

**Figure 2:**
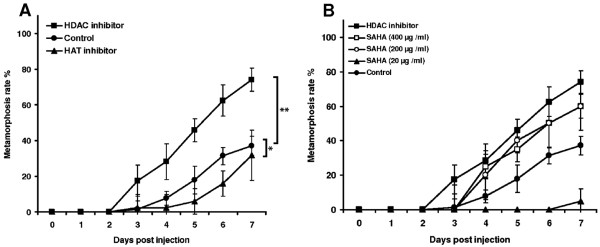
**Effect of HDAC andHAT inhibitors on themetamorphosis of *G. mellonella *larvae.** Last-instar larvae formed a significantly greater number of pupae when injected with 1:1 SAHA (1 mg/ml) and sodium butyrate (20 mg/ml) compared to control larvae treated with 1% DMSO, whereas the injection of HAT inhibitor (500 μg/ml) delayed pupation (**A**). The acceleration of development by inhibition of HDACs was dose-depended as injection of SAHA in lower concentrations (400 μg/ml, 200 μg/ml or 20 μg/ml) resulted in correspondingly reduced acceleration of pupae formation (**B**). The larvae were incubated at 37°C on an artificial diet. Results represent mean values ± standard deviations of at least three independent measurements with 80 animals for HDAC inhibitor, HAT inhibitor or 1% DMSO treatment (*, *p* < 0.05; **, *p* < 0.005) and two experiments with total 10 animals for each concentration.

To test the above hypothesis, we injected specific HDAC and HAT inhibitors into last-instar larvae before pupation. We used a mixture of two HDAC inhibitors, suberoylanilide hydroxamic acid (SAHA) and sodium butyrate, to ensure that the HDAC complex was strongly inhibited. This treatment significantly accelerated pupation compared to control larvae injected with 1% DMSO (Figure 2A), suggesting that histone acetylation increases DNA accessibility and induces the precocious expression of developmentally-regulated genes before pupation. In agreement with this hypothesis, we observed the opposite effect when HAT inhibitors were injected into last-instar larvae. Four and five days post-injection, we observed significantly reduced rates of pupation in injected larvae, but after seven days the differences compared to controls became less remarkable (Figure 2A). To test whether inhibition of HDACs accelerates development of *G. mellonella* in a dose-dependent manner we injected SAHA in different concentrations into last-instar larvae. Indeed, we observed that increasing concentrations of SAHA accelerated correspondingly the formation of pupae (Figure 2B).

### Effects of HAT and HDAC inhibitors on survival following septic injury

**Figure 3:**
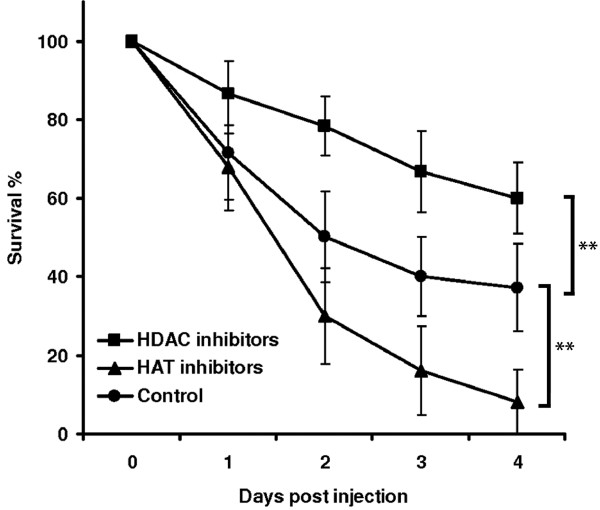
**Effect of HDAC andHAT inhibitors on thesurvival of *G. mellonella *larvae following septic injury.** HDAC inhibitors (1:1 SAHA (1 mg/ml) and sodium butyrate (20 mg/ml)) significantly increased the survival of larvae following injury and hemolymph loss, whereas HAT inhibitor (500 μg/ml) significantly reduced the survival of larvae following injury and hemolymph loss, in each case in comparison to control larvae treated with 1% DMSO. Results represent mean values of at least three independent measurements ± standard deviations from 80 larvae per treatment (**, *p* < 0.005).

The injury of last-instar larvae with a needle caused the loss of hemolymph resulting in 63% mortality after 4 days. In order to determine whether histone acetylation was involved in transcriptional reprogramming following injury, we injected the larvae with specific HDAC or HAT inhibitors prior to wounding. We found that the injection of HDAC inhibitors significantly enhanced survival (only 40% mortality after 4 days), whereas HAT inhibitors had the opposite effect, increasing the mortality to 92% after 4 days (Figure 3).

**Figure 4:**
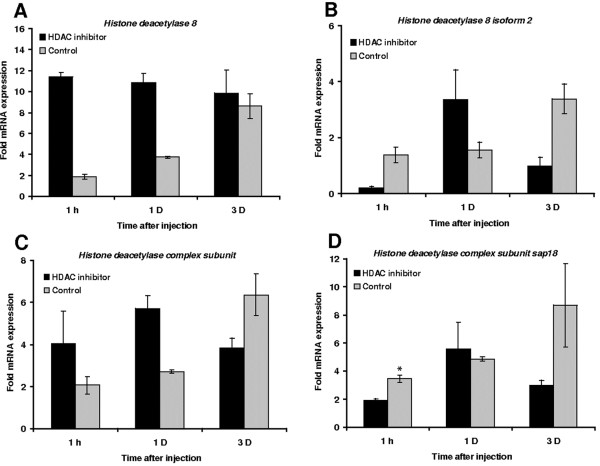
**Transcriptional activation of HDACsafter the administration ofHDAC inhibitors prior toseptic injury.** The transcription of (**A**) *histone deacetylase 8*, (**B**) *histone deacetylase 8 isoform2*, (**C**) *histone deacetylase complex* and (**D**) *histone deacetylase complex subunitsap 18* was measured by quantitative real time RT-PCR following the injection of HDAC inhibitors (1:1 SAHA (1 mg/ml) and sodium butyrate (20 mg/ml)) prior to injury and hemolymph loss, relative to the expression levels in control larvae treated with 1% DMSO. Values were normalized against the 18S rRNA housekeeping gene and represent means of three independent measurements ± standard deviations (*, *p* < 0.05).

**Figure 5:**
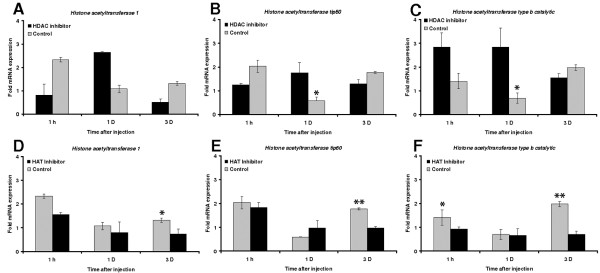
**Transcriptional activation of HATsafter administration of HDACand HAT inhibitors priorto septic injury.** The transcription of *histone acetyltransferase 1*, *histone acetyltransferase tip 60*, and *histone acetyltransferase type bcatalytic subunit* was measured by quantitative real time RT-PCR. The larvae were injected either with (**A-C**) HDAC inhibitors (1:1 SAHA (1 mg/ml) and sodium butyrate (20 mg/ml)) or with (**D-F**) HAT inhibitor (500 μg/ml), prior to injury and hemolymph loss. The expression levels were calculated relative to the expression levels in control larvae treated with 1% DMSO. Values were normalized against the 18S rRNA housekeeping gene and represent means of three independent measurements ± standard deviations (*, *p* < 0.05; **, *p* < 0.005).

We also measured the expression of genes encoding HDACs and HATs at three post-injury time points (1 h, 1 d and 3 d). We found that wounding induced the expression of certain genes encoding HDACs and HATs in control larvae (Figures 4 and 5). The injection of HDAC inhibitors before wounding significantly reduced the expression of *HDAC complex subunit sap18* after 1 hour of injection compared to controls, whereas the expression of the other genes encoding HDACs was induced only transiently compared to controls (Figures 4A-D). HAT expression in larvae treated with HDAC inhibitors was significantly induced for up to 1 day post-injury but after 3 days the levels were suppressed compared to untreated controls (Figures 5A-C).

**Figure 6:**
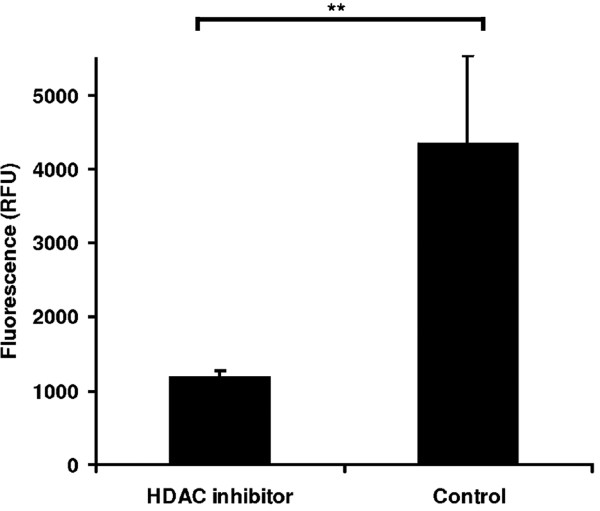
**Measurement of HDAC activityin *G. mellonella *hemocytes following the administrationof HDAC inhibitors.** Hemocytes from injured *Galleria* larvae were seeded in a 96-well clear-bottom black plate at a density of 3 x 10^4^ per well in *Drosophila* Schneider’s medium supplemented with 10% FBS. The cells were treated with 10 μl of HDAC inhibitor cocktail (1:1 SAHA (1 mg/ml) and sodium butyrate (20 mg/ml)) and control hemocytes were treated with an equal volume of 1% DMSO (**, *p* < 0.005).

The wounding of last-instar larvae resulted in the rapid induction of genes encoding HATs. The highest expression levels were observed 1 h post-injury, declining in the following days. The injection of a HAT inhibitor prior to injury dampened this response (Figures 5D-F). To determine whether the attenuated expression of HDAC genes resulted in lower HDAC activity in isolated *G. mellonella* larval hemocytes, we measured the corresponding enzyme activity using an independent method based on photometric fluorescence quantitation. As expected, we found that HDAC inhibitors dampened the induced expression of HDACs in response to injury accompanied with severe hemolymph loss (Figure 6).

### Effects of HDAC inhibitors on gene expression

To investigate the impact of HDAC inhibitors on gene expression induced by wounding, we selected four effectors that were previously found to play essential roles in wound healing and immunity in *G. mellonella*, namely a matrix metalloproteinase (MMP) that exerts pleiotropic functions during metamorphosis and the immune response [12], IMPI, which specifically inhibits microbial metalloproteinases causing sepsis [16,17], p38 MAP kinase, which contributes to immunity-related signaling, and the defensin-like antibacterial peptide galiomycin [15].

**Figure 7:**
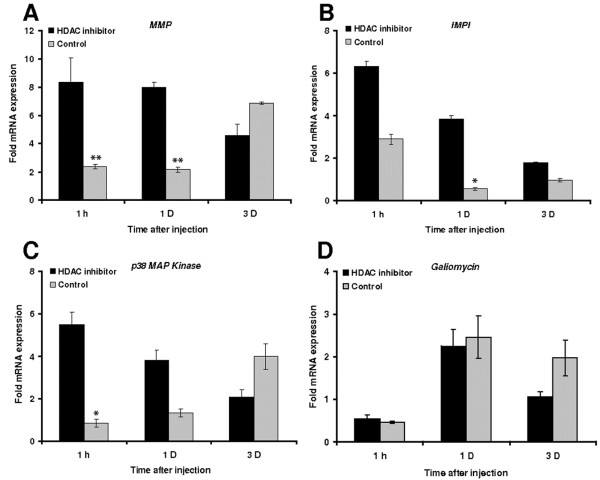
**Transcriptional activation of immune-responsegenes after the administrationof HDAC inhibitors priorto septic injury.** The transcription of (**A**) *MMP*, (**B**) *IMPI*, (**C**) *p38 MAP kinase* and (**D**) *galiomycin* was measured by quantitative real time RT-PCR following the injection of HDAC inhibitors (1:1 SAHA (1 mg/ml) and sodium butyrate (20 mg/ml)) prior to injury and hemolymph loss, compared to control larvae treated with 1% DMSO. Values were normalized against the 18S rRNA housekeeping gene and represent means of three independent measurements ± standard deviations (*, *p* < 0.05; **, p<0.005).

We measured the expression of these genes at the three post-injury time points described above and found that their expression profiles in control larvae were distinct. *MMP* and *p38 MAP kinase* expression increased continuously, whereas *IMPI* expression peaked 1 h after wounding, and *galiomycin* expression peaked 1 d after wounding (Figures 7A-D). In contrast, when larvae were injected with the HDAC inhibitors, *MMP* and *p38 MAP kinase* were significantly induced after 1 h, and IMPI after 1 d with respect to the control. Only *galiomycin* was induced to the same extent in control larvae and those treated with HDAC inhibitors, but the expression of this gene too fell below basal expression levels in the treated larvae after 3 days (Figure 7D).

### Pathogen-induced developmental shifts

**Figure 8:**
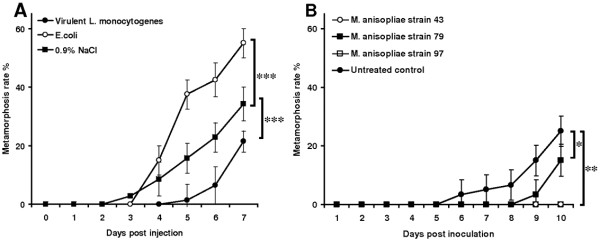
**Metamorphosis of *G. mellonella *larvae following infection withbacterial and fungal pathogens.** (**A**) Injection of pathogenic *L. monocytogenes* or non-pathogenic *E. coli* into last-instar larvae induced opposing developmental effects. Infection with non-pathogenic *E. coli* induced the precocious formation of pupae whereas infection with pathogenic *L. monocytogenes* caused a significant delay in comparison to control larvae treated with 0.9% NaCl. The larvae were incubated at 37°C and reared on an artificial diet. (**B**) Application of conidia from different entomopathogenic *M. anisopliae* strains to the cuticle of last-instar larvae resulted in infection and induced opposing developmental effects. Infection with strains 43 and 97 delayed the formation of pupae whereas infection with strain 79 accelerated pupation. The larvae were incubated at 25°C and reared on an artificial diet. Results represent mean values of at least three independent measurements ± standard deviations from at least 20 larvae per treatment (*, *p* < 0.05; **, *p* < 0.005; ***, *p* < 0.0005).

The infection of last-instar *G. mellonella* larvae with the parasitic fungus *M. anisopliae* strains 43 and 97 or virulent human pathogen *L. monocytogenes* strain EGD-e postponed the formation pupae compared to untreated larvae and those injected with 0.9% NaCl, whereas pupation was accelerated in larvae injected with non-pathogenic *E. coli* (Figures 8A-B).

Furthermore, infection with *M. anisopliae* strains 43, 79 and 97 caused a significant increase in larval mortality compared to uninfected controls (Additional file 1: Figure S1). The opposite developmental effects of virulent and non-virulent microbes described above, combined with previous reports showing developmental and immunological defects resulting from the disruption of HAT and HDAC activities [1-3], led to our hypothesis that pathogens may interfere with histone acetylation and deacetylation to suppress the expression of immunity-related genes in the host, with a collateral impact on the progress of development. To test this hypothesis we quantified expression levels of HATs or HDACs during infection.

### Expression of HAT and HDAC genes during infection

**Figure 9:**
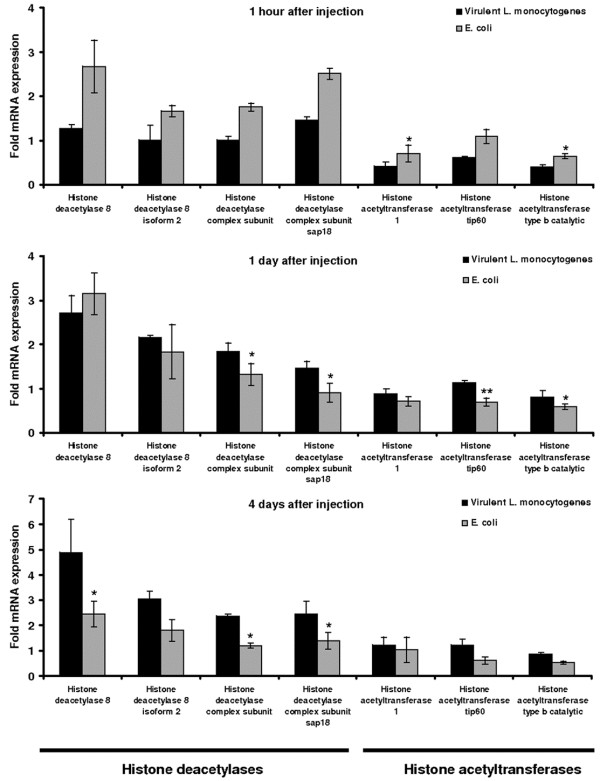
**Transcriptional activation of HDACand HAT genes followingchallenge with pathogenic andnon-pathogenic bacteria.** Transcription levels were measured by quantitative real time RT-PCR (**A**) 1 h, (**B**) 1 d and (**C**) 4 d after infection, and are relative to the levels in larvae treated with 0.9% NaCl. Values were normalized against the 18S rRNA housekeeping gene and represent means of three independent measurements ± standard deviations (*, *p* < 0.05; **, *p* < 0.005).

The simultaneous upregulation of HDAC and HAT genes suggests that normal development requires finely balanced enzyme activities, and that the disruption of this balance should interfere with metamorphosis. We therefore tested the impact of infections with *L. monocytogenes* and *M. anisopliae* on the expression of the seven HAT/HDAC genes discussed above, using real-time RT-PCR to determine the expression levels at three post-infection time points. We found that the HDACs were induced more strongly than HATs throughout the experiment (Figures 9A-C and Additional file 2: Figure S2A-C) and also that the HDAC/HAT balance was restored rapidly in larvae infected with non-pathogenic *E. coli* or *M. anisopliae* strain 79. In contrast, the imbalance persisted significantly for up to 9 days in larvae infected with virulent *L. monocytogenes* in comparison to non-pathogenic *E. coli*. These results suggested that the pathogens interfere with the regulation of histone acetylation and deacetylation in the infected host.

## Discussion

We used the well-established *G. mellonella* model system [6-14] to show that epigenetic reprogramming during insect metamorphosis, wound healing and infection is controlled by histone acetylation/deacetylation, which in turn is regulated by HATs and HDACs with opposing activities. We found that several genes encoding components of HATs and HDACs were upregulated during the transformation of prepupae into pupae (Figure 1). This concurs with previous reports discussing extensive transcriptional reprogramming at the onset of metamorphosis to induce genes involved in tissue and organ remodeling. For example, HAT activity is mediated by the histone H3 acetylase dGN5, which is a key modulator of chromatin structure and transcription during *Drosophila melanogaster* metamorphosis [5].

To provide experimental evidence for the epigenetic role of histone acetylation in *G. mellonella* metamorphosis, we postulated that imbalanced HDAC and HAT activities would either accelerate or postpone development. We therefore injected specific HDAC and HAT inhibitors into last-instar larvae and found that the HDAC inhibitors reduced histone deacetylation following precocious metamorphosis, whereas the HAT inhibitors postponed the formation of pupae (Figure 2A). This, to the best of our knowledge, is the first time that the inhibition of epigenetic regulators with opposing functions has been shown to induce correspondingly opposed developmental effects. Inspired by these results we sought to explore whether histone acetylation also regulates transcriptional reprogramming in response to wounding.

We pierced last-instar *G. mellonella* larvae with a needle to cause injuries accompanied by massive hemolymph loss and tissue damage that was ultimately lethal to the majority of the wounded larvae. We found that the injection of HDAC or HAT inhibitors prior to wounding resulted in opposite effects on mortality compared to control larvae injected with 1% DMSO (Figure 3). These data provide evidence that histone acetylation/deacetylation is also involved in wounding-mediated transcriptional reprogramming. The positive effect of HDAC inhibitors on *G. mellonella* survival after wounding agrees closely with the recently published results of a similar study using the mouse model, in which the HDAC inhibitor SAHA was shown to increase survival in mice suffering from septic shock without fluid resuscitation [20].

The contribution of histone acetylation/deacetylation to wounding-mediated transcriptional reprogramming was investigated in more detail by measuring the expression of genes encoding components of HDACs and HATs identified in our previous transcriptomic analysis of developmental and immunological gene repertoires in *G. mellonella*[14]. Unfortunately, we have not found genes encoding further components of the histone acetylation/deacetylation complex such as SIN3A or SIR2.

As expected, wounding induced the expression of HDACs more than HATs (Figures 4 and 5) indicating that skewing the ratio of HDAC:HAT activity in favor of hypoacetylation ultimately increases the lethality associated with wounding. This is further supported by the observation that the injection of HDAC inhibitors prior to injury results in the accelerated and enhanced expression of both HDACs and HATs (Figures 4 and 5) and thus we hypothesize that larvae with a more balanced HDAC:HAT ratio were more likely to survive. We also studied the effect of HDAC inhibitors on the expression of HDACs using an independent method based on the photometric measurement of enzyme activity. We found that HDAC inhibitors attenuate HDAC level, thus reducing HDAC enzyme activity (Figure 6). Therefore, three lines of evidence indicate a role for histone acetylation in wounding-mediated reprogramming: the opposing effects of HAT and HDAC inhibitors on the survival of wounded larvae, the transcriptional activity of genes encoding HATs and HDACs, and the direct measurement of HDAC activity in the hemolymph.

Because the injection of HDAC inhibitors into last-instar larvae brings forward the onset of pupation, we postulated that the same treatment should enhance and/or bring forward the expression of genes contributing to both tissue remodeling during metamorphosis and wound healing. To test this hypothesis, we measured the expression of the first MMP discovered in Lepidoptera, since this is known to have pleiotropic roles in both development and immunity [18,19]. Confirming our expectations, we found that the *MMP* gene was induced to higher levels following wounding in larvae treated with HDAC inhibitors (Figure 7A). The insect metalloproteinase inhibitor (IMPI), the only animal peptide that specifically inhibits microbial metalloproteases causing sepsis [16,17], was induced 1 h post-injury, but the induction was earlier and stronger in larvae injected with HDAC inhibitors prior to injury (Figure 7B). This may explain the protective effect of HDAC inhibitors following injury accompanied by severe hemolymph loss. The observation that HDAC inhibitors suppress the expression of the antimicrobial peptide *galiomycin* in *G. mellonella* (Figure 7D) agrees with a recent study providing evidence that HDAC inhibitors impair innate immune responses to Toll-like receptor agonists and to infection in mice [21].

The observation that HAT and HDAC inhibitors have opposite effects on development and survival after wounding suggested that histone acetylation/deacetylation may also regulate transcriptional reprogramming during pathogen infections. It was recently reported that *G. mellonella* is an ideal model host for *L. monocytogenes*, a major food-borne human pathogen responsible for listeriosis, which in its severest form can cause fatal meningitis, meningoencephalitis and septicemia [22,23]. The injection of *G. mellonella* larvae with bacterial LPS significantly enhanced survival rates upon subsequent infection with a nominally lethal dose of *L. monocytogenes* by inducing the expression of antimicrobial peptides that have now been characterized [9]. We found that interactions between *G. mellonella* and virulent strains of *L. monocytogenes* caused a delay in the transformation of infected larvae into pupae (Figure 9). We therefore postulated that at least this pathogen can interfere with histone acetylation in the host because infection causes the same developmental delay that we observed when larvae were injected with HAT inhibitors (Figure 2). These findings agree with recent experiments showing that *L. monocytogenes* can also delay the induction of host cellular immunity in mammalian hosts to prolong the infection cycle [24]. Furthermore, immune stimulation also accelerates the development of the red flour beetle *Tribolium castaneum* following a challenge with heat-killed bacteria, although links to epigenetic gene regulation have not been explored [25]. Interestingly, we have also observed accelerated pupation in *G. mellonella* larvae following treatment with heat-killed *L. monocytogenes* (data not shown).

The ability of virulent *L. monocytogenes* and *M. anisopliae* strains to interfere with epigenetic gene regulation and exert codependent depressive effects on immunity and development led to a further hypothesis that non-virulent microbes lacking the ability to suppress the immune system might have a collateral accelerating effect on development. We therefore administered *G. mellonella* larvae with non-pathogenic *E. coli* or non-pathogenic *M. anisopliae* strain. As postulated, these infections had the opposite effect to infections with virulent pathogens, not only failing to delay metamorphosis but actually accelerating the process (Figure 8), as observed when larvae were injected with HDAC inhibitors (Figure 2).

The hemolysin known as listeriolysin O (LLO) has previously been shown to inhibit histone acetylation and phosphorylation in human epithelial cells infected with *L. monocytogenes*[26,27], whereas prolonged exposure to LPS and infection with pathogenic *Mycobacterium tuberculosis* strains causes the transcriptional induction of multiple HDAC genes in mammals [28]. Bacteria have therefore been shown to induce chromatin remodeling during infection, although their impact on host immunity and development is unclear. Our data provide the first conclusive evidence that pathogens can interfere with the epigenetic regulation of transcription to influence both immunity and development in insects. We found that infection with non-pathogenic *E. coli* induced HDAC gene expression after 1 h but that the levels were attenuated after 4 d, whereas infection with virulent *L. monocytogenes* resulted in the delayed but longer-lasting induction of HDACs, particularly HDAC8 (Figure 9). In contrast, HAT expression levels were not significantly affected at the later time point, indicating that the opposing developmental effects caused by virulent and non-virulent bacteria are predominantly mediated through the regulation of HDAC activity. Similarly, infection with entomopathogenic *M. anisopliae* strain 43 and 97 resulted in early mortality and late pupation than strain 79. We have recorded early induction of HDACs (2 and 4 days after infection) in larvae infected with *M. anisopliae* strain 43 and 97 but not with the strain 79. The skewing HDAC/HAT ratio following *M. anisopliae* strain 79 infection was apparently distinct only 9 days after infection, indicating the differences in the pathogenic potential of fungal strains in causing mortal infections. The proposed role of HDACs as a regulatory layer of immunity-related signaling in insects may be evolutionarily conserved [29] because our data are consistent with recent findings showing that the mouse metastatic tumor antigen 1 (MTA1) coregulator, a component of the nucleosome remodeling and deacetylase (NuRD) complex, modulates the NF-κB signaling network to maintain homeostasis during inflammatory responses [30].

## Conclusions

In conclusion, we found that last-instar *G. mellonella* larvae underwent early metamorphosis compared to controls when injected with HDAC inhibitors or non-pathogenic *E. coli* and *M. anisopliae* strains*,* whereas those injected with HAT inhibitors or virulent *L. monocytogenes* and *M. anisopliae* strains showed a developmental delay compared to controls. We observed corresponding shifts in both HDAC expression and activity in the hemolymph. These data taken together support our hypothesis that histone acetylation regulates epigenetic transcriptional reprogramming during metamorphosis, following wounding and during infection with virulent bacteria in insects. In addition, pathogens such as *L. monocytogenes* can manipulate histone acetylation in the infected host, potentially to suppress immune responses, but through the resulting transcriptional reprogramming this has a collateral impact on development. Further research will focus on identifying acetylated histones and detecting their individual role during metamorphosis, wounding and infection.

## Materials and methods

### Insect rearing and bacterial and fungal cultures

*G. mellonella* larvae were reared at 32°C in darkness and on an artificial diet (22% maize meal, 22% wheat germ, 11% dry yeast, 17.5% bees wax, 11% honey, 11% glycerin). Last-instar larvae, each weighing 250–350 mg, were used in all experiments.

*L. monocytogenes* strain EGD-e (serotype 1/2a) was cultured aerobically in Brain Heart Infusion (BHI) medium (Difco, Franklin Lakes, NY) at 37°C and on BHI agar plates. Non-pathogenic *E. coli* strain K12 was grown in LB medium at 37°C and on LB agar plates. For injection experiments, we used logarithmic phase bacterial cultures (10^9^ cfu/ml in 10 ml BHI or LB broth) after washing and serial dilution in 0.9% NaCl. We injected 10 μl of the cultures (10^8^ cfu/ml) dorsolaterally into the hemocoel of last-instar larvae using 1-ml disposable syringes and 0.4 × 20 mm needles mounted on a micromanipulator.

The parasitic fungus *M. anisopliae* was obtained from the Julius-Kühn-Institute in Darmstadt (Germany). The fungal cultures were grown on potato dextrose agar (Carl Roth, Germany) at 27°C for 10 days to initiate conidiogenesis. Last-instar larvae were inoculated with isolated conidia at a concentration of 3000 conidia/ml to replicate a natural infection.

### Treatment with HAT/HDAC inhibitors

HDAC inhibitor SAHA (Cayman Chemicals, Estonia) and the HAT inhibitor Epigenetic Multiple Ligand (Merck, Darmstadt, Germany) were separately dissolved in 1% DMSO (Carl Roth, Germany) whereas the HDAC inhibitor sodium butyrate (Sigma Aldrich, Germany) was dissolved in sterile water. SAHA (1 mg/ml) and sodium butyrate (20 mg/ml) were mixed in a 1:1 ratio and 20 μl of the combination was injected dorsolaterally into the hemocoel of unisexual last-instar larvae using 1-ml disposable syringes and 0.4- by 20-mm needles mounted on a microapplicator. The same volume of HAT inhibitor (500 μg/ml) was injected. Control larvae were injected with 1% DMSO. One hour after treatment the larvae were injured with a sterile needle causing severe hemorrhage and hemolymph loss. The larvae were incubated at 37°C with food and the mortality rates were recorded.

### RT-PCR

Five larvae per treatment for each time point were homogenized in 1 ml Trizol reagent (Sigma Aldrich), and whole RNA was extracted according to the manufacturer's recommendations. The isolated RNA was subjected to DNase digestion (Qiagen) to eliminate traces of DNA, if present. The RNA integrity was confirmed by nanodrop with 260/280 absorbance of ~2.0. Complementary DNA synthesis was performed using the First Stand cDNA synthesis kit (Fermentas). The quantity of RNA and cDNA was determined spectrophotometrically and the integrity was confirmed by ethidium bromide gel staining. Quantitative real time RT-PCR was performed with the Biorad real-time PCR system (CFX 96) using the SsoFast EvaGreen Supermix protocol (Biorad). We used 50 ng of cDNA per reaction to amplify the HAT and HDAC genes using the primers shown in Table 1. The PCR programme used for gene amplification includes initial activation at 95°C for 10 mins, denaturation at 95°C for 15 seconds, annealing at 56°C for 15 seconds and extension at 72°C for 15 seconds. This programme was repeated for 39 cycles.

### Measurement of HDAC activity

HDAC activity was monitored using a cell-based assay kit (Enzol Life Sciences, Exeter, UK). Briefly, *G. mellonella* hemocytes were cultured in *Drosophila* Schneider’s medium (supplemented with 10% FBS) in microtiter plates and washed with HDAC assay buffer (Enzol Life Sciences) following the addition of HDAC inhibitors SAHA (1 mg/ml) and sodium butyrate (20 mg/ml) mixed in a 1:1 ratio. Control hemocytes were treated with 1% DMSO. HDAC activity in *G. mellonella* hemocytes was initiated by adding HDAC substrate to each well. The cells were incubated at 37°C for 2 h and Lysis/Developer solution was added to each well and incubated at 37°C for 15 min. The fluorescence intensity was measured using a Tecan plate reader (excitation wavelength 340–360 nm, emission wavelength 440–460 nm).

### Data analysis

All experiments were performed a minimum of three times. Significant differences between values were compared using a Student's *t*-test, with a significance threshold of p < 0.05.

## Abbreviations

HDAC: histone deacetylase; HAT: Histone acetyltransferase; IMPI: Insect Metalloproteinase Inhibitor; MAP: Mitogen-activated protein; RT-PCR: Reverse transcriptase polymerase chain reaction; SAHA: Suberoylanilide hydroxamic acid; MMP: Matrix metalloproteinase; BHI: Brain heart infusion; LB: Luria broth.

## Competing interests

The authors’ declare that they have no competing interests.

## Authors’ contributions

AV conceived the study. KM performed the experimental work. KM, RF and AV contributed to data analysis and drafted the manuscript. All authors contributed in the conception and design of the study, read and approved the final version of the manuscript.

## Supplementary Material

Additional file 1 **Figure S1.** Survival of *G. mellonella* larvae following inoculation with different *M. anisopliae* strains. The application of *M. anisopliae* conidia resulted in the infection of *G. mellonella* larvae. Infection with *M. anisopliae* strains 43 (○) and 97 (□) significantly increased the larval mortality compared to the untreated control (●), whereas infection with strain 79 (■) significantly reduced the mortality rate. Results represent mean values of at least three independent experiments ± standard deviations from at least 20 larvae per treatment (*, *p* < 0.05; **, *p* < 0.005; ***, *p* < 0.0005). Click here for file

Additional file 2 **Figure S2.** Transcriptional activation of HDAC and HAT genes following infection with pathogenic fungi. Transcription levels following infection by the entomopathogenic *M. anisopliae* strains 43, 79 and 97, were measured by quantitative real time RT-PCR **(A)** 2 d, **(B)** 4 d and **(C)** 9 d after infection, and are relative to the levels in untreated larvae. Values were normalized against the 18S rRNA housekeeping gene and represent means of three independent measurements ± standard deviations. Click here for file
