# Supplementary figures and images for: Histone acetylation mediates epigenetic regulation of transcriptional reprogramming in insects during metamorphosis, wounding and infection
Source: Front Zool. 2012 Oct 4;9:25. doi: 10.1186/1742-9994-9-25 (PMC3538701; doi:10.1186/1742-9994-9-25)

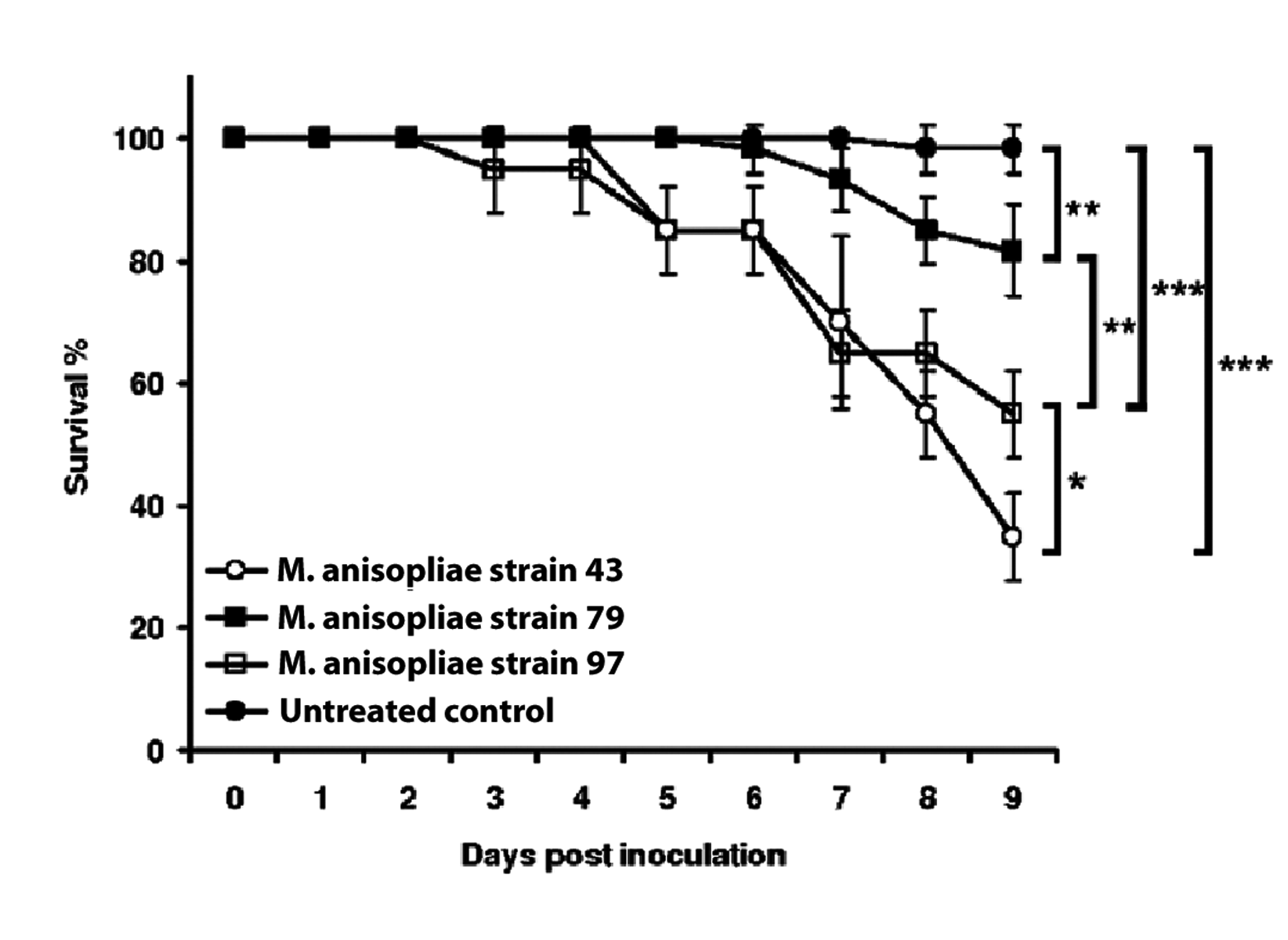

Supplement: Additional file 1 — Figure S1. Survival of G. mellonella larvae following inoculation with different M. anisopliae strains. The application of M. anisopliae conidia resulted in the infection of G. mellonella larvae. Infection with M. anisopliae strains 43 (○) and 97 (□) significantly increased the larval mortality compared to the untreated control (●), whereas infection with strain 79 (■) significantly reduced the mortality rate. Results represent mean values of at least three independent experiments ± standard deviations from at least 20 larvae per treatment (*, p < 0.05; **, p < 0.005; ***, p < 0.0005). [file 1742-9994-9-25-S1.tiff]

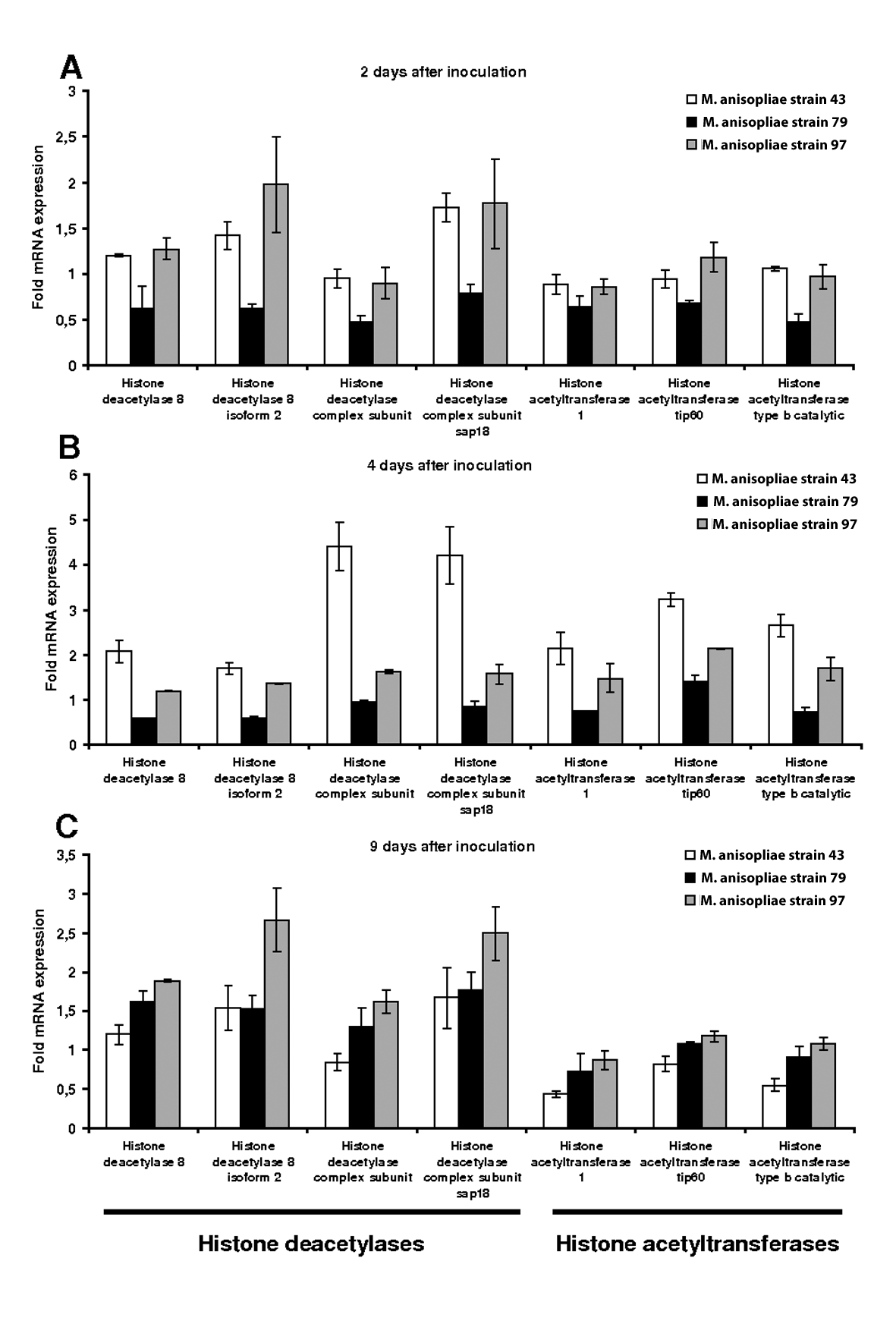

Supplement: Additional file 2 — Figure S2. Transcriptional activation of HDAC and HAT genes following infection with pathogenic fungi. Transcription levels following infection by the entomopathogenic M. anisopliae strains 43, 79 and 97, were measured by quantitative real time RT-PCR (A) 2 d, (B) 4 d and (C) 9 d after infection, and are relative to the levels in untreated larvae. Values were normalized against the 18S rRNA housekeeping gene and represent means of three independent measurements ± standard deviations. [file 1742-9994-9-25-S2.tiff]
